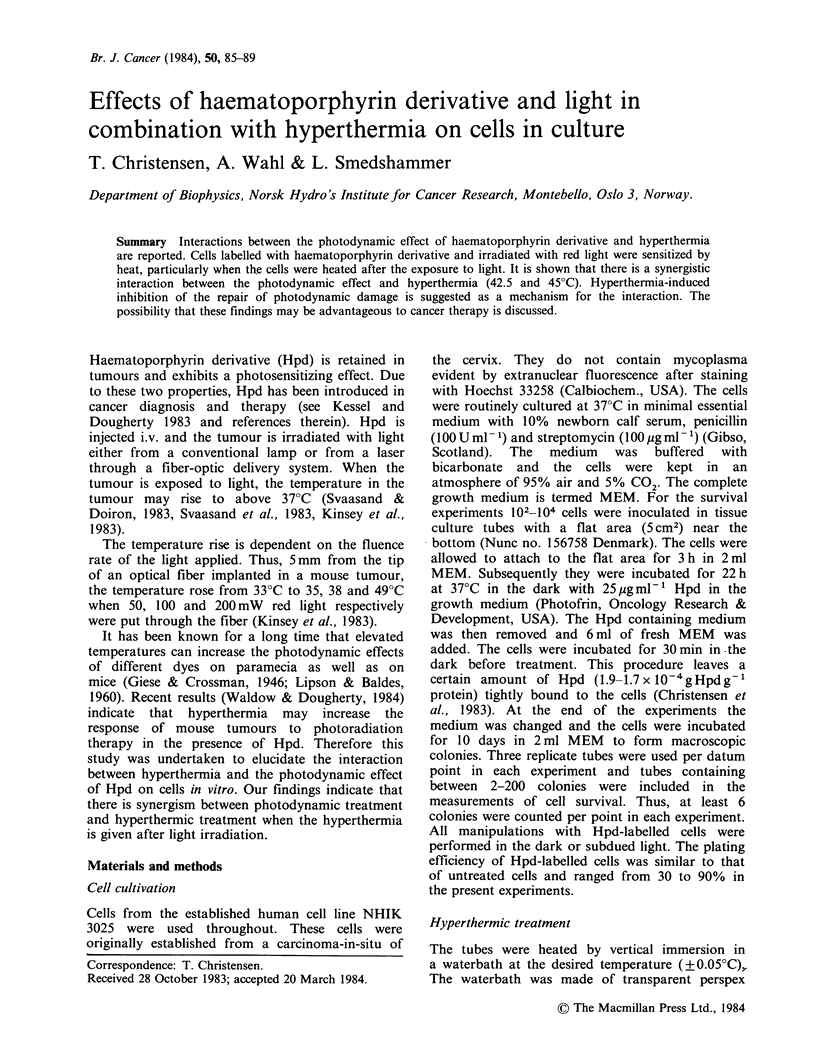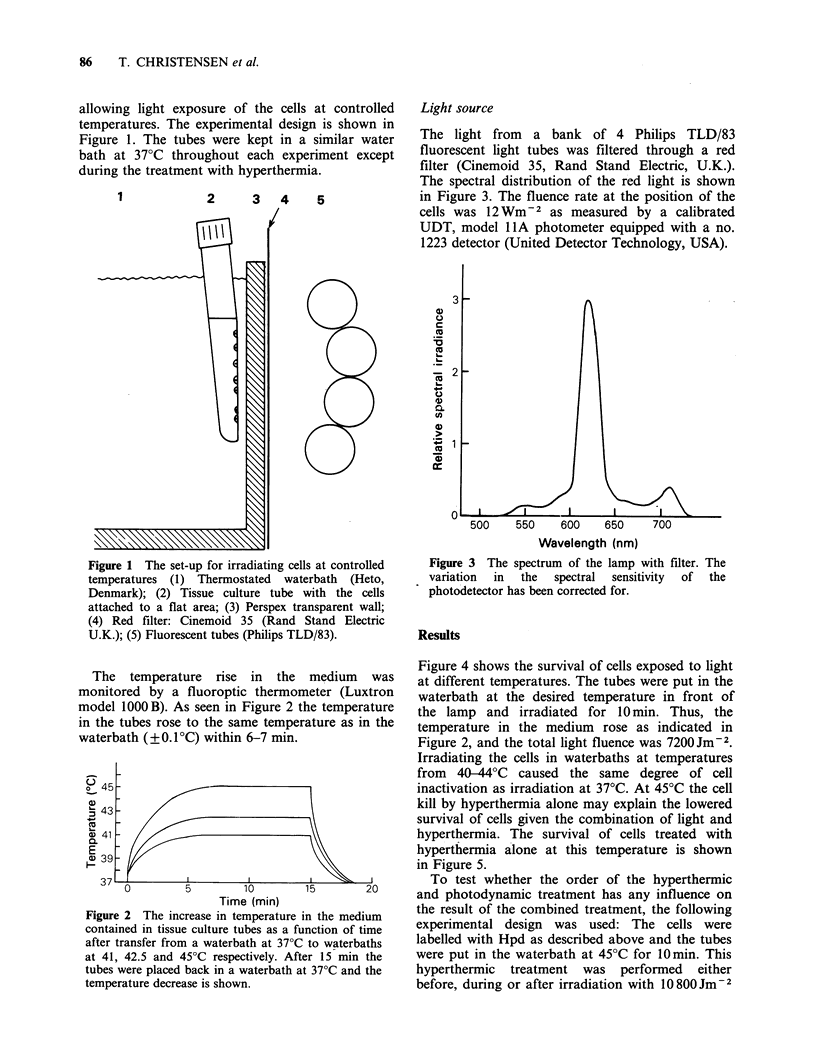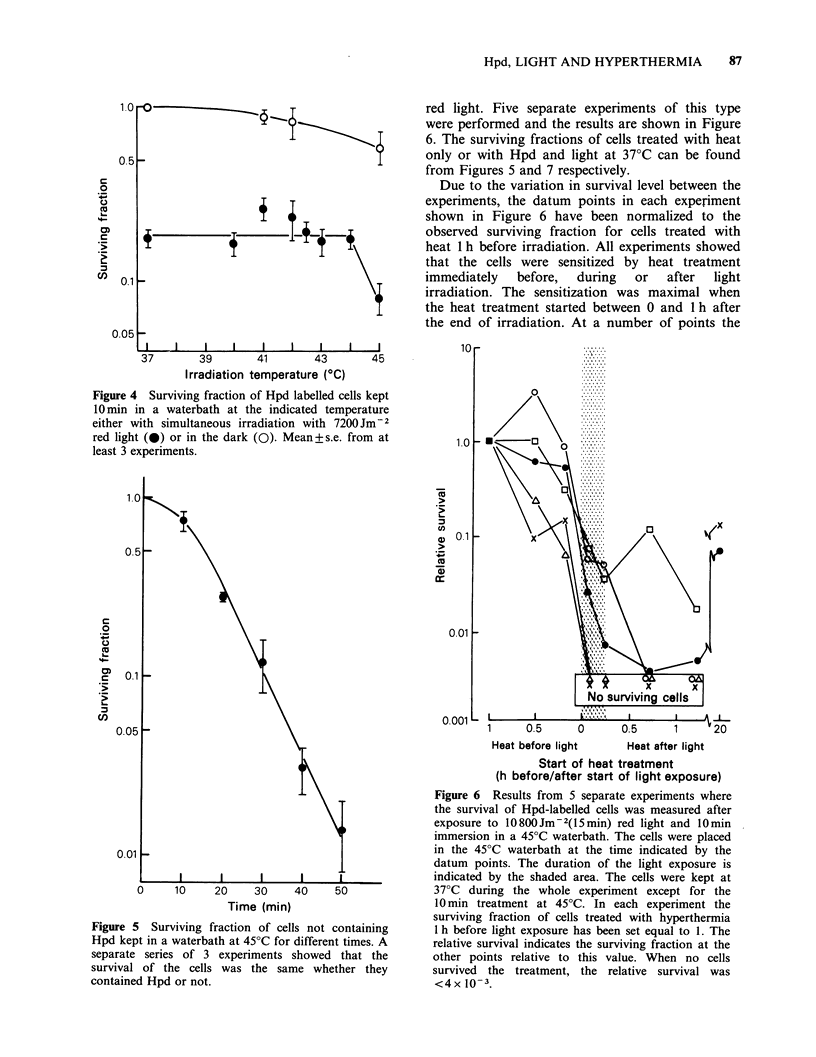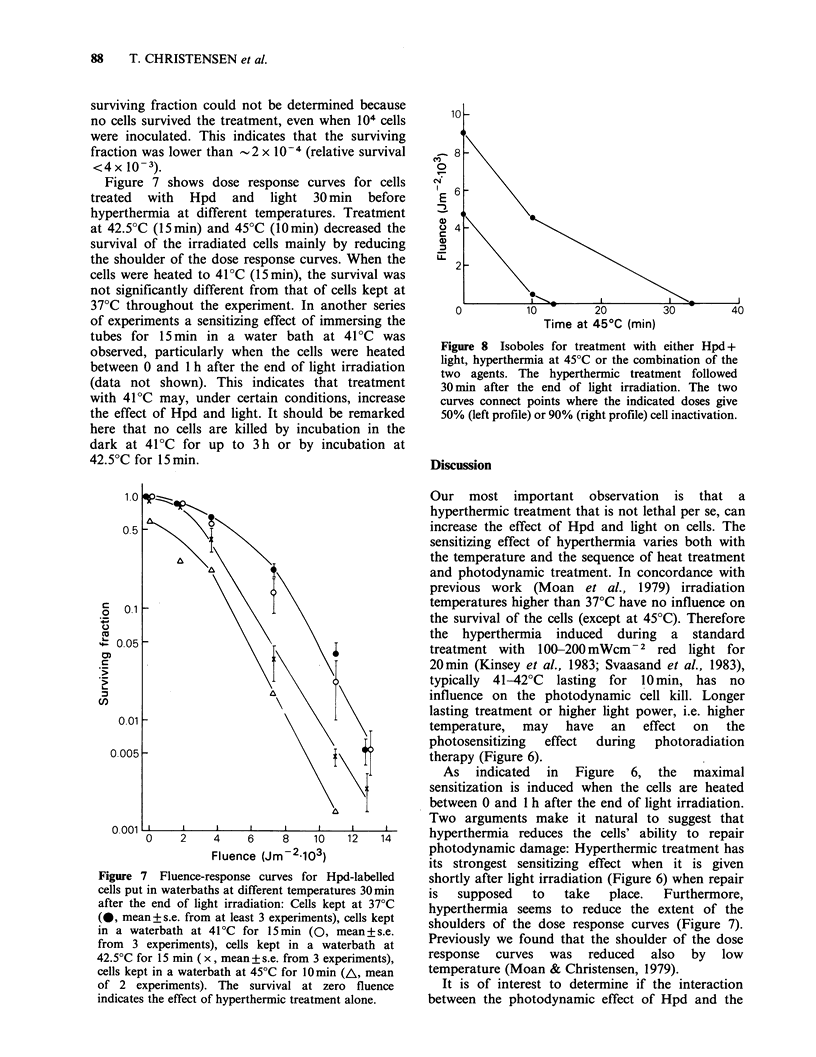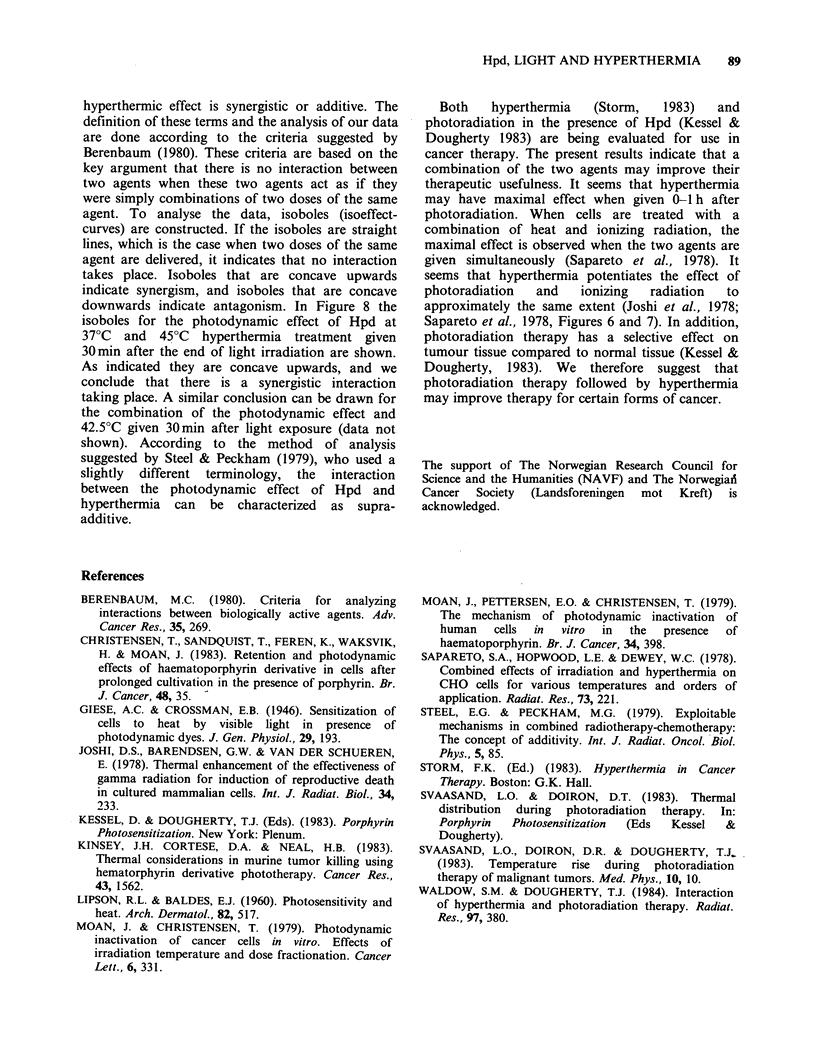# Effects of haematoporphyrin derivative and light in combination with hyperthermia on cells in culture.

**DOI:** 10.1038/bjc.1984.142

**Published:** 1984-07

**Authors:** T. Christensen, A. Wahl, L. Smedshammer

## Abstract

Interactions between the photodynamic effect of haematoporphyrin derivative and hyperthermia are reported. Cells labelled with haematoporphyrin derivative and irradiated with red light were sensitized by heat, particularly when the cells were heated after the exposure to light. It is shown that there is a synergistic interaction between the photodynamic effect and hyperthermia (42.5 and 45 degrees C). Hyperthermia-induced inhibition of the repair of photodynamic damage is suggested as a mechanism for the interaction. The possibility that these findings may be advantageous to cancer therapy is discussed.


*Br. J. Cancer* (1984), **50**, 85–89

### Effects of haematoporphyrin derivative and light in combination with hyperthermia on cells in culture

T. Christensen, A. Wahl & L. Smedshammer

*Department of Biophysics, Norsk Hydro's Institute for Cancer Research, Montebello, Oslo 3, Norway.*

**Summary** Interactions between the photodynamic effect of haematoporphyrin derivative and hyperthermia are reported. Cells labelled with haematoporphyrin derivative and irradiated with red light were sensitized by heat, particularly when the cells were heated after the exposure to light. It is shown that there is a synergistic interaction between the photodynamic effect and hyperthermia (42.5 and 45°C). Hyperthermia-induced inhibition of the repair of photodynamic damage is suggested as a mechanism for the interaction. The possibility that these findings may be advantageous to cancer therapy is discussed.

Haematoporphyrin derivative (Hpd) is retained in tumours and exhibits a photosensitizing effect. Due to these two properties, Hpd has been introduced in cancer diagnosis and therapy (see Kessel and Dougherty 1983 and references therein). Hpd is injected i.v. and the tumour is irradiated with light either from a conventional lamp or from a laser through a fiber-optic delivery system. When the tumour is exposed to light, the temperature in the tumour may rise to above 37°C (Svaasand & Doiron, 1983, Svaasand *et al.*, 1983, Kinsey *et al.*, 1983).

The temperature rise is dependent on the fluence rate of the light applied. Thus, 5 mm from the tip of an optical fiber implanted in a mouse tumour, the temperature rose from 33°C to 35, 38 and 49°C when 50, 100 and 200 mW red light respectively were put through the fiber (Kinsey *et al.*, 1983).

It has been known for a long time that elevated temperatures can increase the photodynamic effects of different dyes on paramecia as well as on mice (Giese & Crossman, 1946; Lipson & Baldes, 1960). Recent results (Waldow & Dougherty, 1984) indicate that hyperthermia may increase the response of mouse tumours to photoradiation therapy in the presence of Hpd. Therefore this study was undertaken to elucidate the interaction between hyperthermia and the photodynamic effect of Hpd on cells *in vitro*. Our findings indicate that there is synergism between photodynamic treatment and hyperthermic treatment when the hyperthermia is given after light irradiation.

### Materials and methods

### *Cell cultivation*

Cells from the established human cell line NHIK 3025 were used throughout. These cells were originally established from a carcinoma-in-situ of the cervix. They do not contain mycoplasma evident by extranuclear fluorescence after staining with Hoechst 33258 (Calbiochem., USA). The cells were routinely cultured at 37°C in minimal essential medium with 10% newborn calf serum, penicillin (100 U ml$^{-1}$) and streptomycin (100 $\mu$g ml$^{-1}$) (Gibso, Scotland). The medium was buffered with bicarbonate and the cells were kept in an atmosphere of 95% air and 5% $CO_2$. The complete growth medium is termed MEM. For the survival experiments $10^2$–$10^4$ cells were inoculated in tissue culture tubes with a flat area (5 cm$^2$) near the bottom (Nunc no. 156758 Denmark). The cells were allowed to attach to the flat area for 3 h in 2 ml MEM. Subsequently they were incubated for 22 h at 37°C in the dark with 25 $\mu$g ml$^{-1}$ Hpd in the growth medium (Photofrin, Oncology Research & Development, USA). The Hpd containing medium was then removed and 6 ml of fresh MEM was added. The cells were incubated for 30 min in the dark before treatment. This procedure leaves a certain amount of Hpd (1.9–1.7 $\times 10^{-4}$ g Hpd g$^{-1}$ protein) tightly bound to the cells (Christensen *et al.*, 1983). At the end of the experiments the medium was changed and the cells were incubated for 10 days in 2 ml MEM to form macroscopic colonies. Three replicate tubes were used per datum point in each experiment and tubes containing between 2–200 colonies were included in the measurements of cell survival. Thus, at least 6 colonies were counted per point in each experiment. All manipulations with Hpd-labelled cells were performed in the dark or subdued light. The plating efficiency of Hpd-labelled cells was similar to that of untreated cells and ranged from 30 to 90% in the present experiments.

### *Hyperthermic treatment*

The tubes were heated by vertical immersion in a waterbath at the desired temperature ($\pm$0.05°C). The waterbath was made of transparent perspex

Correspondence: T. Christensen.
Received 28 October 1983; accepted 20 March 1984.

© The Macmillan Press Ltd., 1984

allowing light exposure of the cells at controlled temperatures. The experimental design is shown in Figure 1. The tubes were kept in a similar water bath at 37°C throughout each experiment except during the treatment with hyperthermia.

**Figure 1** The set-up for irradiating cells at controlled temperatures (1) Thermostated waterbath (Heto, Denmark); (2) Tissue culture tube with the cells attached to a flat area; (3) Perspex transparent wall; (4) Red filter: Cinemoid 35 (Rand Stand Electric U.K.); (5) Fluorescent tubes (Philips TLD/83).

The temperature rise in the medium was monitored by a fluoroptic thermometer (Luxtron model 1000 B). As seen in Figure 2 the temperature in the tubes rose to the same temperature as in the waterbath (±0.1°C) within 6–7 min.

**Figure 2** The increase in temperature in the medium contained in tissue culture tubes as a function of time after transfer from a waterbath at 37°C to waterbaths at 41, 42.5 and 45°C respectively. After 15 min the tubes were placed back in a waterbath at 37°C and the temperature decrease is shown.

*Light source*

The light from a bank of 4 Philips TLD/83 fluorescent light tubes was filtered through a red filter (Cinemoid 35, Rand Stand Electric, U.K.). The spectral distribution of the red light is shown in Figure 3. The fluence rate at the position of the cells was $12\,Wm^{-2}$ as measured by a calibrated UDT, model 11A photometer equipped with a no. 1223 detector (United Detector Technology, USA).

**Figure 3** The spectrum of the lamp with filter. The variation in the spectral sensitivity of the photodetector has been corrected for.

### Results

Figure 4 shows the survival of cells exposed to light at different temperatures. The tubes were put in the waterbath at the desired temperature in front of the lamp and irradiated for 10 min. Thus, the temperature in the medium rose as indicated in Figure 2, and the total light fluence was $7200\,Jm^{-2}$. Irradiating the cells in waterbaths at temperatures from 40–44°C caused the same degree of cell inactivation as irradiation at 37°C. At 45°C the cell kill by hyperthermia alone may explain the lowered survival of cells given the combination of light and hyperthermia. The survival of cells treated with hyperthermia alone at this temperature is shown in Figure 5.

To test whether the order of the hyperthermic and photodynamic treatment has any influence on the result of the combined treatment, the following experimental design was used: The cells were labelled with Hpd as described above and the tubes were put in the waterbath at 45°C for 10 min. This hyperthermic treatment was performed either before, during or after irradiation with $10\,800\,Jm^{-2}$

**Figure 4** Surviving fraction of Hpd labelled cells kept 10 min in a waterbath at the indicated temperature either with simultaneous irradiation with 7200 $Jm^{-2}$ red light (●) or in the dark (○). Mean ± s.e. from at least 3 experiments.

**Figure 5** Surviving fraction of cells not containing Hpd kept in a waterbath at 45°C for different times. A separate series of 3 experiments showed that the survival of the cells was the same whether they contained Hpd or not.

red light. Five separate experiments of this type were performed and the results are shown in Figure 6. The surviving fractions of cells treated with heat only or with Hpd and light at 37°C can be found from Figures 5 and 7 respectively.

Due to the variation in survival level between the experiments, the datum points in each experiment shown in Figure 6 have been normalized to the observed surviving fraction for cells treated with heat 1 h before irradiation. All experiments showed that the cells were sensitized by heat treatment immediately before, during or after light irradiation. The sensitization was maximal when the heat treatment started between 0 and 1 h after the end of irradiation. At a number of points the

**Figure 6** Results from 5 separate experiments where the survival of Hpd-labelled cells was measured after exposure to 10 800 $Jm^{-2}$(15 min) red light and 10 min immersion in a 45°C waterbath. The cells were placed in the 45°C waterbath at the time indicated by the datum points. The duration of the light exposure is indicated by the shaded area. The cells were kept at 37°C during the whole experiment except for the 10 min treatment at 45°C. In each experiment the surviving fraction of cells treated with hyperthermia 1 h before light exposure has been set equal to 1. The relative survival indicates the surviving fraction at the other points relative to this value. When no cells survived the treatment, the relative survival was $<4\times10^{-3}$.

surviving fraction could not be determined because no cells survived the treatment, even when $10^4$ cells were inoculated. This indicates that the surviving fraction was lower than $\sim 2\times10^{-4}$ (relative survival $<4\times10^{-3}$).

Figure 7 shows dose response curves for cells treated with Hpd and light 30 min before hyperthermia at different temperatures. Treatment at 42.5°C (15 min) and 45°C (10 min) decreased the survival of the irradiated cells mainly by reducing the shoulder of the dose response curves. When the cells were heated to 41°C (15 min), the survival was not significantly different from that of cells kept at 37°C throughout the experiment. In another series of experiments a sensitizing effect of immersing the tubes for 15 min in a water bath at 41°C was observed, particularly when the cells were heated between 0 and 1 h after the end of light irradiation (data not shown). This indicates that treatment with 41°C may, under certain conditions, increase the effect of Hpd and light. It should be remarked here that no cells are killed by incubation in the dark at 41°C for up to 3 h or by incubation at 42.5°C for 15 min.

**Figure 7** Fluence-response curves for Hpd-labelled cells put in waterbaths at different temperatures 30 min after the end of light irradiation: Cells kept at 37°C (●, mean ± s.e. from at least 3 experiments), cells kept in a waterbath at 41°C for 15 min (○, mean ± s.e. from 3 experiments), cells kept in a waterbath at 42.5°C for 15 min (×, mean ± s.e. from 3 experiments), cells kept in a waterbath at 45°C for 10 min (△, mean of 2 experiments). The survival at zero fluence indicates the effect of hyperthermic treatment alone.

**Figure 8** Isoboles for treatment with either Hpd + light, hyperthermia at 45°C or the combination of the two agents. The hyperthermic treatment followed 30 min after the end of light irradiation. The two curves connect points where the indicated doses give 50% (left profile) or 90% (right profile) cell inactivation.

### Discussion

Our most important observation is that a hyperthermic treatment that is not lethal per se, can increase the effect of Hpd and light on cells. The sensitizing effect of hyperthermia varies both with the temperature and the sequence of heat treatment and photodynamic treatment. In concordance with previous work (Moan *et al.*, 1979) irradiation temperatures higher than 37°C have no influence on the survival of the cells (except at 45°C). Therefore the hyperthermia induced during a standard treatment with 100–200 mWcm$^{-2}$ red light for 20 min (Kinsey *et al.*, 1983; Svaasand *et al.*, 1983), typically 41–42°C lasting for 10 min, has no influence on the photodynamic cell kill. Longer lasting treatment or higher light power, i.e. higher temperature, may have an effect on the photosensitizing effect during photoradiation therapy (Figure 6).

As indicated in Figure 6, the maximal sensitization is induced when the cells are heated between 0 and 1 h after the end of light irradiation. Two arguments make it natural to suggest that hyperthermia reduces the cells' ability to repair photodynamic damage: Hyperthermic treatment has its strongest sensitizing effect when it is given shortly after light irradiation (Figure 6) when repair is supposed to take place. Furthermore, hyperthermia seems to reduce the extent of the shoulders of the dose response curves (Figure 7). Previously we found that the shoulder of the dose response curves was reduced also by low temperature (Moan & Christensen, 1979).

It is of interest to determine if the interaction between the photodynamic effect of Hpd and the hyperthermic effect is synergistic or additive. The definition of these terms and the analysis of our data are done according to the criteria suggested by Berenbaum (1980). These criteria are based on the key argument that there is no interaction between two agents when these two agents act as if they were simply combinations of two doses of the same agent. To analyse the data, isoboles (isoeffect-curves) are constructed. If the isoboles are straight lines, which is the case when two doses of the same agent are delivered, it indicates that no interaction takes place. Isoboles that are concave upwards indicate synergism, and isoboles that are concave downwards indicate antagonism. In Figure 8 the isoboles for the photodynamic effect of Hpd at 37°C and 45°C hyperthermia treatment given 30 min after the end of light irradiation are shown. As indicated they are concave upwards, and we conclude that there is a synergistic interaction taking place. A similar conclusion can be drawn for the combination of the photodynamic effect and 42.5°C given 30 min after light exposure (data not shown). According to the method of analysis suggested by Steel & Peckham (1979), who used a slightly different terminology, the interaction between the photodynamic effect of Hpd and hyperthermia can be characterized as supra-additive.

Both hyperthermia (Storm, 1983) and photoradiation in the presence of Hpd (Kessel & Dougherty 1983) are being evaluated for use in cancer therapy. The present results indicate that a combination of the two agents may improve their therapeutic usefulness. It seems that hyperthermia may have maximal effect when given 0–1 h after photoradiation. When cells are treated with a combination of heat and ionizing radiation, the maximal effect is observed when the two agents are given simultaneously (Sapareto *et al.,* 1978). It seems that hyperthermia potentiates the effect of photoradiation and ionizing radiation to approximately the same extent (Joshi *et al.,* 1978; Sapareto *et al.,* 1978, Figures 6 and 7). In addition, photoradiation therapy has a selective effect on tumour tissue compared to normal tissue (Kessel & Dougherty, 1983). We therefore suggest that photoradiation therapy followed by hyperthermia may improve therapy for certain forms of cancer.

The support of The Norwegian Research Council for Science and the Humanities (NAVF) and The Norwegian Cancer Society (Landsforeningen mot Kreft) is acknowledged.

### References

BERENBAUM, M.C. (1980). Criteria for analyzing interactions between biologically active agents. *Adv. Cancer Res.,* **35,** 269.

CHRISTENSEN, T., SANDQUIST, T., FEREN, K., WAKSVIK, H. & MOAN, J. (1983). Retention and photodynamic effects of haematoporphyrin derivative in cells after prolonged cultivation in the presence of porphyrin. *Br. J. Cancer,* **48,** 35.

GIESE, A.C. & CROSSMAN, E.B. (1946). Sensitization of cells to heat by visible light in presence of photodynamic dyes. *J. Gen. Physiol.,* **29,** 193.

JOSHI, D.S., BARENDSEN, G.W. & VAN DER SCHUEREN, E. (1978). Thermal enhancement of the effectiveness of gamma radiation for induction of reproductive death in cultured mammalian cells. *Int. J. Radiat. Biol.,* **34,** 233.

KESSEL, D. & DOUGHERTY, T.J. (Eds). (1983). *Porphyrin Photosensitization.* New York: Plenum.

KINSEY, J.H. CORTESE, D.A. & NEAL, H.B. (1983). Thermal considerations in murine tumor killing using hematorphyrin derivative phototherapy. *Cancer Res.,* **43,** 1562.

LIPSON, R.L. & BALDES, E.J. (1960). Photosensitivity and heat. *Arch. Dermatol.,* **82,** 517.

MOAN, J. & CHRISTENSEN, T. (1979). Photodynamic inactivation of cancer cells *in vitro.* Effects of irradiation temperature and dose fractionation. *Cancer Lett.,* **6,** 331.

MOAN, J., PETTERSEN, E.O. & CHRISTENSEN, T. (1979). The mechanism of photodynamic inactivation of human cells *in vitro* in the presence of haematoporphyrin. *Br. J. Cancer,* **34,** 398.

SAPARETO, S.A., HOPWOOD, L.E. & DEWEY, W.C. (1978). Combined effects of irradiation and hyperthermia on CHO cells for various temperatures and orders of application. *Radiat. Res.,* **73,** 221.

STEEL, E.G. & PECKHAM, M.G. (1979). Exploitable mechanisms in combined radiotherapy-chemotherapy: The concept of additivity. *Int. J. Radiat. Oncol. Biol. Phys.,* **5,** 85.

STORM, F.K. (Ed.) (1983). *Hyperthermia in Cancer Therapy.* Boston: G.K. Hall.

SVAASAND, L.O. & DOIRON, D.T. (1983). Thermal distribution during photoradiation therapy. In: *Porphyrin Photosensitization* (Eds Kessel & Dougherty).

SVAASAND, L.O., DOIRON, D.R. & DOUGHERTY, T.J. (1983). Temperature rise during photoradiation therapy of malignant tumors. *Med. Phys.,* **10,** 10.

WALDOW, S.M. & DOUGHERTY, T.J. (1984). Interaction of hyperthermia and photoradiation therapy. *Radiat. Res.,* **97,** 380.